# Mental Health Sequelae in Health Professionals in Spain during the COVID Pandemic

**DOI:** 10.1192/j.eurpsy.2022.90

**Published:** 2022-09-01

**Authors:** P. Lusilla-Palacios

**Affiliations:** Vall d’Hebron University Hospital-VHIR Autonomous University of Barcelona, Department Of Psychiatry, Barcelona, Spain

**Keywords:** Covid-19, healthcare workers, physician health programs, Mental Disorders

## Abstract

The COVID-19 pandemic has raised several concerns regarding its mental health effect on patients and professionals. In the beginning, the absence of knowledge about the disease transmission or effective therapies, the quick spread among the population collapsing hospitals in combination with the lack of protection measures put healthcare professionals working in the frontline in a high stressful situation. The professionals had to face several unprecedented challenges: improvised hospitals, living in hotels to avoid infecting the family, deciding, as in wartime, which patients could be intubated and which could not, doubling shifts, and above all, the uncertainty about the disease, the high severity and the contagiousness that isolated the patients from their family, leaving the health professional with the responsibility of being a caregiver in the broad sense of the word. With this picture several studies have reported a high prevalence of mental disorders. A survey of 9138 Spanish professionals conducted during the first wave of the pandemic showed that 45.7% had a mental disorder (depression, generalized anxiety disorder, panic attacks, post-traumatic stress disorder and SUD), 14.5% had any disabling current mental disorder and 8.4% had suicidal thoughts. In Spain, managed by the Galatea Foundation, there is a special programme of confidential care for doctors with a mental illness or addiction. During the pandemic, a 30% increase of requests for help were registered, 70% of which came from primary health care professionals. The presentation provides also qualitative data with testimonies of professionals and anti-stress protection measures implemented by some health institutions.

**Disclosure:**

No significant relationships.

